# Near obstacles detection by inverse perspective mapping of AVM for intelligent vehicles

**DOI:** 10.1371/journal.pone.0336851

**Published:** 2026-01-05

**Authors:** Zhe Zhou, Yule Liao, Bolong Wang, Minwang Wang, Min Fu, Zhaozheng Hu

**Affiliations:** 1 Hubei Key Laboratory of Vehicle-Infrastructure Collaboration and Traffic Control, Hubei University of Arts and Science, Xiangyang, China; 2 Hubei Key Laboratory of Transportation Internet of Things, Wuhan University of Technology, Wuhan, China; 3 Intelligent Transportation Systems Research Center, Wuhan University of Technology, Wuhan, China; UCSF: University of California San Francisco, UNITED STATES OF AMERICA

## Abstract

Although state-of-the-art sensors, such as LiDAR, radar, monocular camera, along with detection algorithms for intelligent vehicles, generally exhibit superior performance in object detection and recognition, they still encounter significant challenges in detecting near obstacles due to the blind sensing areas. To address this issue, we propose a near obstacle detection method named NOD-AVM (near object detection based on around view monitoring), which utilizes the four wide-angle cameras of the AVM. From the four wide-angle cameras of the AVM, a total of four NOD-AVMs were developed, whose sensing areas are the intersections of two adjacent cameras. In the context of NOD-AVMs, the application of the inverse perspective mapping (IPM) is used to project images from adjacent cameras onto the ground plane. By analyzing the difference between the two adjacent IPM images, the system can ascertain the presence of obstacles on the ground plane. Once an obstacle is detected, the IPM image also allows us to estimate the distance with respect to the ego-vehicle. To validate the feasibility and effectiveness of the proposed NOD-AVM, we have conducted experiments using real-world data collected by a prototype intelligent vehicle in both campus and urban road environments. Experimental results demonstrate that the proposed method can efficiently detect both static and dynamic obstacles near the ego-vehicle and accurately locate them. Dataset and code were uploaded as Support information.

## 1. Introduction

As a crucial function of autonomous vehicles, obstacle detection is among the most essential components in ADAS (advanced driver assistance systems) and autonomous driving systems. Any obstacle detection malfunctions can lead to catastrophic accidents [1]. Consequently, intelligent vehicles must accurately detect and locate nearby obstacles to ensure safe autonomous operation and road safety. The increasing attention to the development of robust obstacle detection systems has become a focal point in the advancement of autonomous driving technology, as evidenced by the progress in sensor integration, data fusion, and deep learning applications [2,3].

In recent years, the field of obstacle detection has seen exceptional progress, with numerous methods being proposed and implemented. Based on the sensing modality, these methods are generally classified into two categories: active sensor-based methods and passive sensor-based methods [4]. Active sensors measure the distances between the sensor and surrounding objects by emitting signals (e.g., electromagnetic waves, acoustic waves) and analyzing their time of flight, such as sonar, radar and LiDAR. In contrast, passive sensors, such as optical and infrared cameras, detect electromagnetic radiation already existing in the environment [5]. Methods based on active sensors try to reconstruct an accurate 3D structure of the environment surrounding the ego-vehicle to facilitate obstacle detection. Among them, ultrasonic radars are widely deployed in mass-produced vehicles for obstacle warning due to their low cost and ease of integration [6,7]. However, the fatal drawback of ultrasonic radars is that the limited scanning range is on a 2D plane rather than 3D space, which leads to significant blind spots. Compared with ultrasonic radars, millimeter-wave radars offer longer detection ranges and greater robustness in adverse weather conditions, thereby enhancing environment perception [8]. Nonetheless, they suffer from a limited field of view (FOV) and are relatively expensive. Standard LiDAR models, such as the HDL-64L, use an array of rotating laser beams to generate 3D point clouds with a 360-degree horizontal FOV and a sensing range of up to 120 meters. This capability has led to their widespread adoption in intelligent vehicle applications [9,10]. Ming et al proposed a road modeling method based on the Markov random field for tiny obstacle detection, which was compatible with different terrains and different LiDARs [11]. However, LiDAR systems still exhibit blind spots, particularly for near obstacle detection. Furthermore, due to their high cost, 3D LiDAR sensors are not well suited for large-scale deployment in intelligent vehicle systems.

Recent advancements in deep learning have significantly improved the performance of obstacle detection and classification. For example, binocular cameras are instrumental in deriving disparity maps via image matching, which subsequently facilitate the generation of detailed 3D reconstructions of the surrounding environment [12]. Based on the 3D reconstruction results, obstacle detection and location estimation become feasible [13]. However, dense 3D reconstruction from stereo vision is usually time-consuming, which hardly satisfies the real-time requirements of intelligent vehicle applications. In practical on-road driving scenarios, the primary objects of interest include motor vehicles, non-motor vehicles, and pedestrians. Numerous detection algorithms, relying solely on monocular camera, have been developed. For instance, Zhang et al. proposed a cross-view consistency 3D keypoint learning method, which leverages relative transformation constraints between different viewpoints to maintain the consistency of keypoints across views, thereby enabling 6D pose estimation [14]. Convolutional neural network (CNN), a cornerstone of deep learning, has significantly advanced visual tasks such as object detection and semantic segmentation, especially in complex environments [15,16]. For example, R-CNN has demonstrated outstanding capabilities in identifying vehicles, pedestrians, and other objects [17]. More advanced CNN architectures have also been proposed, incorporating multi-scale feature integration to further enhance detection accuracy and reliability [18]. Based on these detection results, the location of obstacles can be inferred. However, these obstacle detection methods necessitate extensive and costly data annotation for training datasets and demand significant computational resources. Moreover, these models may fail to detect potentially hazardous objects if such classes were not enumerated beforehand during the training phase. To address these challenges, recent unsupervised learning methods have been inspired by traditional stereo matching techniques, training models with stereo image pairs to estimate depth from a single image [19]. Since accurate ground truth data cannot be obtained, the resulting depth estimations often exhibit uncertainty, particularly at greater distances.

In addition, multi-sensor fusion can compensate for the limitations of individual sensors by enabling cooperative detection across a broader range of scenarios. [20,21]. Nevertheless, this approach necessitates the calibration of heterogeneous sensors, a process that is inherently complex. Furthermore, the calibration accuracy significantly influences the detection outcomes. Currently, AVM systems with a large FOV, compared to the conventional monocular cameras have been extensively adopted in production vehicles for enhanceing driver awareness of surrounding traffic environments [22,23]. Kim et al. applied semantic segmentation to AVM images and utilized evidence theory to improve the segmentation performance [24]. However, the evidence filtering process demands considerable computational resources, encompassing inference and secondary processing threads, rendering it unsuitable for large-scale commercial applications. Furthermore, existing sensor systems and detection algorithms tend to focus on identifying distant objects, frequently neglecting the significance of detecting nearby obstacles. In fact, obstacles typically located within one to two meters of the vehicle are crucial for ensuring the safety of intelligent vehicles, necessitating highly reliable and precise detection. Unfortunately, these obstacles often lie within the blind spots of conventional sensors, posing a significant threat to safe operation.

**Fig 1:**
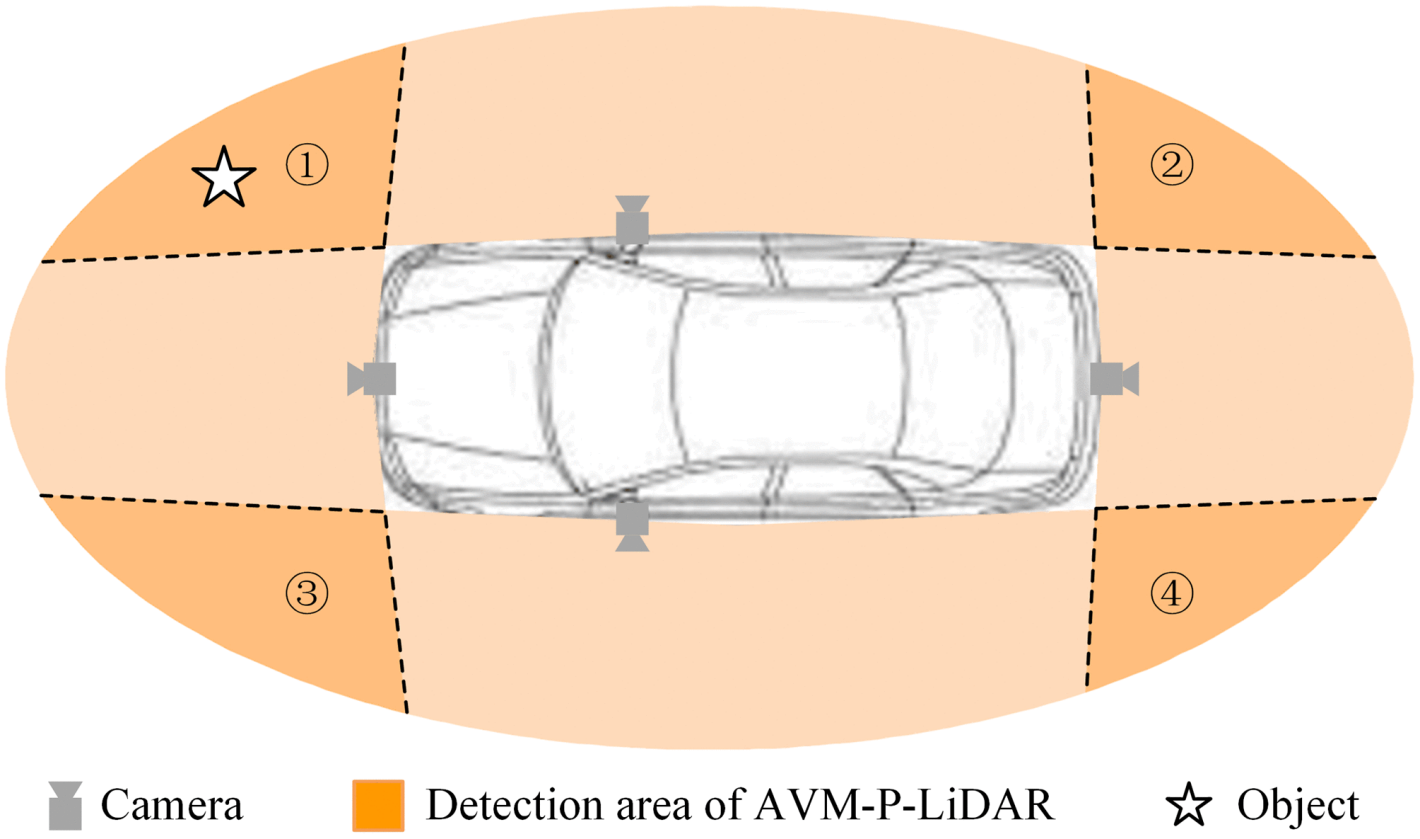
Schematic of NOD-AVM (dark regions (① ~ ④): detection areas of four NOD-AVMs based on AVM cameras).

In this study, we put forward a novel obstacle detection approach named NOD-AVM, which utilizes the in-vehicle AVM system to accomplish near obstacle detection without the need for equipping additional sensors. As shown by the dark regions in **Fig 1**, NOD-AVM detects the presence of obstacles and estimates the location of obstacles relative to the ego-vehicle within the overlapping area. The core principle entails projecting images from different wide-angle cameras

onto a reference plane (e.g., the ground plane) using a homography matrix. By employing back-projection and mutual subtraction techniques, we generate an IPM difference map. Based on the fundamental assumption that pixel values corresponding to the same physical point across different views remain identical, points on the reference plane display zero intensity in the IPM difference map, while off-ground points in the overlapping areas result in non-zero values. Based on this principle, NOD-AVM facilitates accurate obstacle detection. Based on the principle, the proposed NOD-AVM can realize accurate obstacles detection. The main contributions of the proposed NOD-AVM method are summarized as follows:

1) A method for detecting near obstacles in intelligent vehicles is proposed. This method solely utilizes an off-the-shelf AVM to conduct near obstacle detection and high-precision localization without the need for additional hardware, thus resolving the blind-spot issue of conventional sensors.
2) By only implementing basic image processing operations, including calibration, undistortion, binarization, morphological filtering, and thresholding, the proposed detection method not only enhances the performance of near obstacle detection but also considerably reduces the processing time, presenting substantial advantages for intelligent vehicle applications.

To delineate the proposed methodology, the subsequent sections of this paper are structured as follows. Section 2 elaborates on the proposed approach for near obstacle detection and localization. Section 3 depicts the experimental configuration and outcomes. Lastly, Section 4 presents the conclusions of this study.

## 2. The proposed methods

**Fig 2:**
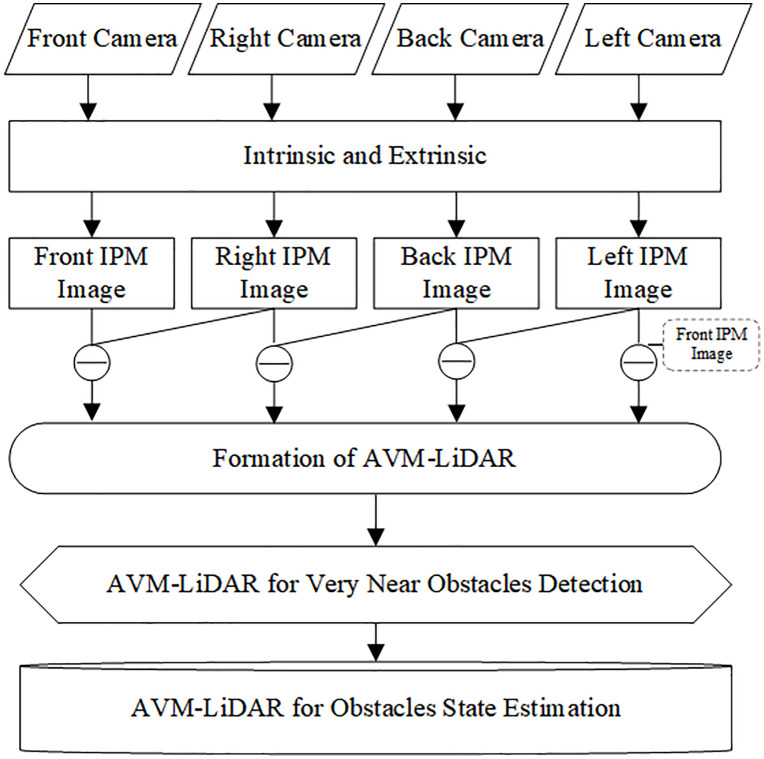
Flowchart of NOD-AVM model for near obstacle detection and localization.

This section elaborates on the NOD-AVM framework designed for NOD and localization. As **Fig 2** is the case, the system consists of four stages: 1) Generating IPM images via camera calibration; 2) Constructing the NOD-AVM model; 3) Near obstacle detection through the NOD-AVM; 4) Obstacle localization estimation. To elucidate the operating principle, we concentrate on the overlapping region between the front and right cameras (i.e., area ① in **Fig 1**) for a detailed elucidation.

### 2.1 AVM calibration and IPM image generation

Let $X=(x,y,z{)}^{T}$ represent the homogeneous coordinates of a point within the ground coordinate system. Its corresponding coordinates in the camera coordinate system are denoted as $\overset{-}{X}=(\overset{-}{x},\overset{-}{y},\overset{-}{z}{)}^{T}$, and its image coordinates as $U=(u,v,1{)}^{T}$. The relationship between X and $\overset{-}{X}$ can be formulated as follows:


$$\overset{-}{X}=RX+t$$ (1)

where ***R*** is the rotation matrix and ***t*** is the translation vector. Collectively, they constitute the extrinsic matrix of the camera. In accordance with the pinhole imaging principle, the mapping relationship between $\overset{-}{X}$ and U can be obtained as:


$$U=\frac{\text{1}}{\overset{-}{z}}K\overset{-}{X}$$ (2)

where 𝐊 denotes the intrinsic matrix of the camera, including focal length, principal point, and skew coefficient. This matrix can be pre-computed using Zhang’s method [25]. Zhang’s method. By detecting the corners of the pattern (typically a checkerboard) and solving a set of homographies, this method estimates both the intrinsic parameters of the camera and the lens distortion coefficients.

Define π as a reference physical plane (i.e., the ground plane in this paper) with the unit normal vector 𝐧 and the distance d from the origin in the ground coordinate system. Based on the spatial relationship between points and planes, it can be inferred that:


$$n^{T}X=d\Rightarrow \frac{n^{T}}{d}X=1$$ (3)

From equations (1–3), we can obtain that:


$$\begin{array}{c} U=\frac{K}{\overset{-}{z}}(R+\frac{n^{T}}{d})X\\ whereH=\frac{K}{\overset{-}{z}}(R+\frac{n^{T}}{d}) \end{array}$$ (4)

The homography matrix is a non-singular 3 × 3 matrix, which is pivotal in linear algebra and matrix transformations. So it can be rewritten as


$$\left[\begin{array}{c} {}*20cu\\ v\\ 1 \end{array}\right]≅s𝐇\left[\begin{array}{c} {}*20cx\\ y\\ 1 \end{array}\right]=\left(\begin{array}{ccc} {}*20ch_{1} & h_{2} & h_{3}\\ h_{4} & h_{5} & h_{6}\\ h_{7} & h_{8} & h_{9} \end{array}\right)\left[\begin{array}{c} {}*20cx\\ y\\ 1 \end{array}\right]$$ (5)

where ***s*** represent the scale factor between the image plane and the ground plane. Given the assumption that the ground is planar, the z-axis is zero, and it is a two-dimensional homogeneous coordinate. By setting the ground plane as the reference plane, IPM images can be generated using the homography matrix ***H***, which is calculated by selected at least four pairs of corresponding points between the reference plane and the image plane. Since homography mapping eliminates perspective distortion, metric measurements, such as computing the distance between objects and the camera, become achievable in the IPM domain. Fisheye cameras demonstrate significant nonlinear distortion, rendering distortion rectification via warping requisite before IPM generation. This rectification employs the nonlinear coefficients acquired from intrinsic calibration. As AVM system is a commercial application, a detailed account of this process is omitted. Readers interested in more details can refer to [26,27].

### 2.2 NOD-AVM modeled from adjacent cameras

Specifically, subsequent to the generation of IPM images, all pixels from the front and right cameras are projected onto the ground plane. Owing to the wide FOV of the cameras (185 degrees each), an overlapping region exists between the IPM images captured by the front and right cameras, as depicted in Fig 4B. The intersection area, which is marked by a red wireframe, serves as the detection region. This region is determined by the camera’s FOVs and installation positions. Given the 185-degree FOV of each camera, the horizontal detection range is approximately 6 meters, the longitudinal range is around 5.2 meters, and the FOV of the overlapping area is approximately 75 degrees. Additionally, motion blur is another factor that requires consideration. Collectively, these factors render our method suitable for low-speed scenarios.

**Fig 3:**
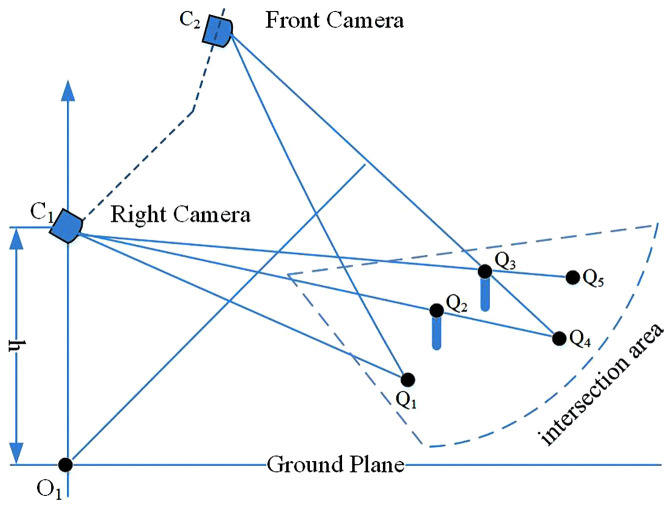
Illustration of inverse perspective mapping (IPM) for off-ground and on-ground objects.

In the IPM images, each physical point within the overlapping area corresponds to two pixel intensities: one from $I_{F}^{\prime }(x,y)$ and the other from $I_{R}^{\prime }(x,y)$. Due to varying viewing angles and installation positions, adjacent images inevitably display disparities in white balance and exposure. Moreover, motion blur can deteriorate the system’s performance. To alleviate these effects, we adopt the method from [28] to reduce motion blur and the method from [29] for intensity normalization. Consequently, the IPM difference map d(x,y) is calculated as follows


$$d(x,y)=|I_{F}^{\prime }(x,y)-I_{R}^{\prime }(x,y)|$$ (6)

In this study, it is postulated that the intensities of pixels corresponding to the same physical point, subsequent to perspective transformation, exhibit identical values. This postulation is substantiated by the following rationales: (1) **Camera consistency**: All cameras utilized in the AVM system are configured with nearly identical imaging parameters to minimize inter-camera disparities. This configuration is in line with the standard practices of typical AVM systems. (2) **Intensity normalization**: An intensity normalization procedure is applied to all images of the AVM, further reducing the discrepancies arising from different cameras and viewpoints. (3) **Fundamental computer vision principle**: This postulation parallels the fundamental premise commonly adopted in classical optical flow methods [30], which assume that correctly matched pixels across two image planes have identical intensity values. The key difference in our approach is the utilization of two distinct cameras capturing spatially different viewpoints, whereas optical flow typically involves a single camera capturing images at temporally different instants. Consequently, within the IPM framework, all pixels mapped onto the ground plane are assumed to have zero intensity difference. As depicted in **Fig 3**, consider point Q_1_ situated on the ground plane, Its projection in an image from camera C_1_ and its projection in an image from camera C_2_ are precisely mapped to the same point (Q_1_) on the ground plane. In this situation, the intensity difference at Q_1_ in the difference map d(x,y) is zero. Any deviation from this would signify an incorrect correspondence during the mapping process. Conversely, for the off-ground point Q_3_, its inverse projection point is Q_4_ in the image of camera C_2_, and Q_5_ in the image of camera C_1_. The intensity difference for Q_3_ in the difference map d(x,y) is significantly different. This principle enables us to determine whether physical points lie on the ground plane. However, due to factors such as vehicle vibration or uneven pavement, the intensity differences of ground points are not always zero. To mitigate the influence of noise, the proposed method tolerates minor variations by employing the threshold defined in (5), rather than demanding an absolute zero difference.


$$\tau (x,y)=\{\begin{array}{c} {}*20c1\;\;if\;\;d(x,y)>\varepsilon \\ 0\;\;if\;\;d(x,y)\leq \varepsilon \end{array}$$ (7)

where τ(x,y) is the binary image generated from the IPM difference map through a threshold ε that adapts to the light intensity and is determined by the method in [31]. Moreover, cameras equipped with additional light sources can also ensure consistent brightness.

**Fig 4:**
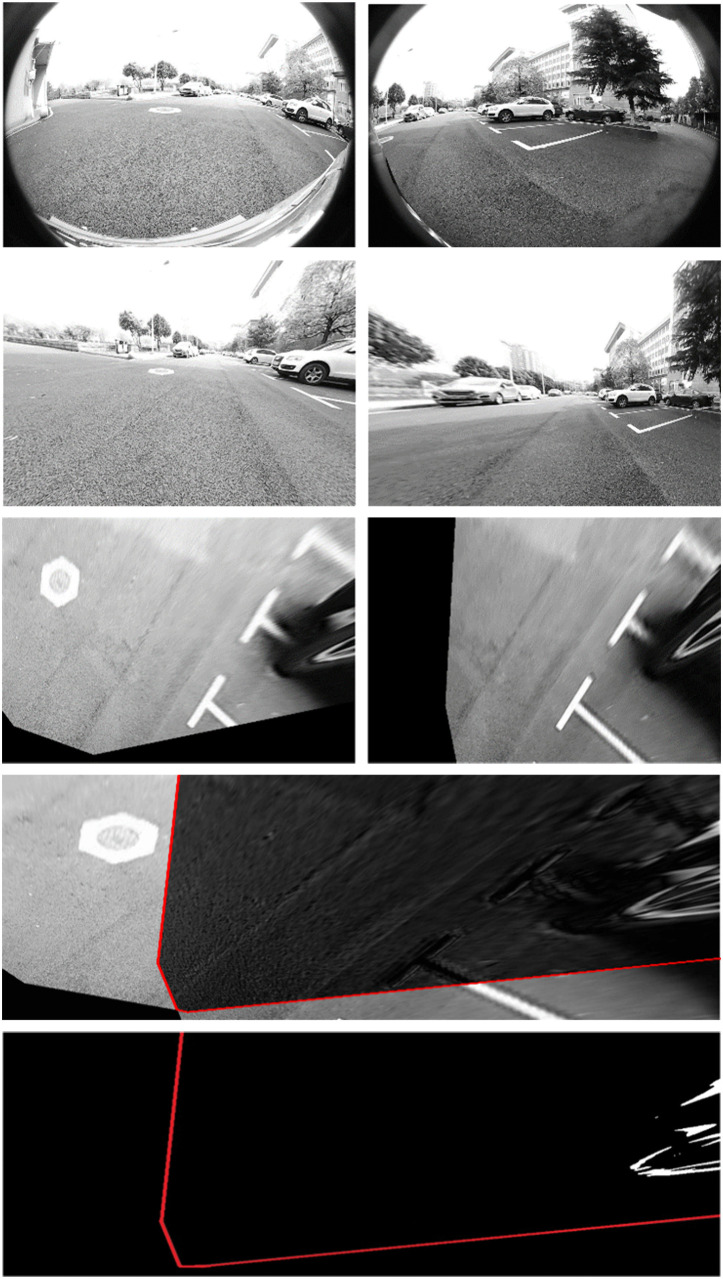
Illustration of NOD-AVM formation process: (A) raw images; (B) undistorted images; (C) IPM images; (D) Projection of corresponding points onto ground plane; (E) final binary image(red polygon: detection area, red rectangle: detection obstacles).

In practice, thresholding may still result in noise. For instance, the pixel value differences from off-ground points (such as Q_2_ and Q_3_, which share the same inverse projection point Q_4_) can be zero or approximately zero, leading the difference map to approach zero in these regions. Nevertheless, these off-ground points with zero or near-zero differences tend to be isolated on the difference map and can thus be eliminated by morphological operations. In contrast, on-ground points generally form connected regions on the difference map, clearly differentiating them from the isolated noise. For further refinement, we suggest employing the opening morphological operation for noise removal, as described below:


b(x,y)=τ(x,y)⊕SE (8)

where *SE* is the element structure for the opening operation defined in(8). The opening operation can effectively remove the isolated noises, significantly enhancing the system’s robustness. The structure element is defined empirically with the size (3, 3). The detection result in the absence of obstacles is shown in **Fig 4**.

### 2.3 Obstacle region extraction

Based on the method outlined in subsection 2.2, images captured by multiple cameras are projected onto the ground plane. Subsequent analysis involves comparing pixel value differences to ascertain whether corresponding points lie on the ground plane. To enhance detection efficiency, the detection scope is constrained by defining a region of interest on IPM images, enabling the identification of ground plane points as detailed below:


$$(x,y)=\left\{ \begin{array}{c} \text{1}\;\;\;(x,y)\;\;on\;the\;ground\\ \text{0}\;\;\;(x,y)\;\;out\;of\;the\;ground \end{array}\;(x,y)\in ROI\right.\;$$ (9)

Moreover, to enhance robustness, we calculate the number of on-ground points N as follows:


N=∑b(x,y) (10)

Hence, obstacles within the overlapping region (denoted by the dark area in **Fig 1**) can be identified by applying a threshold. In contrast to 3D reconstruction techniques, the proposed method achieves obstacle detection utilizing only basic image processing operations, including normalization, homography-based perspective projection, image difference, morphological operations, and thresholding. Consequently, this approach offers high detection accuracy and computational efficiency. Furthermore, it overcomes the limitation of blind spots for near-field obstacles inherent in existing sensor systems.

### 2.4 Obstacle’s location estimation using NOD-AVM

**Fig 5:**
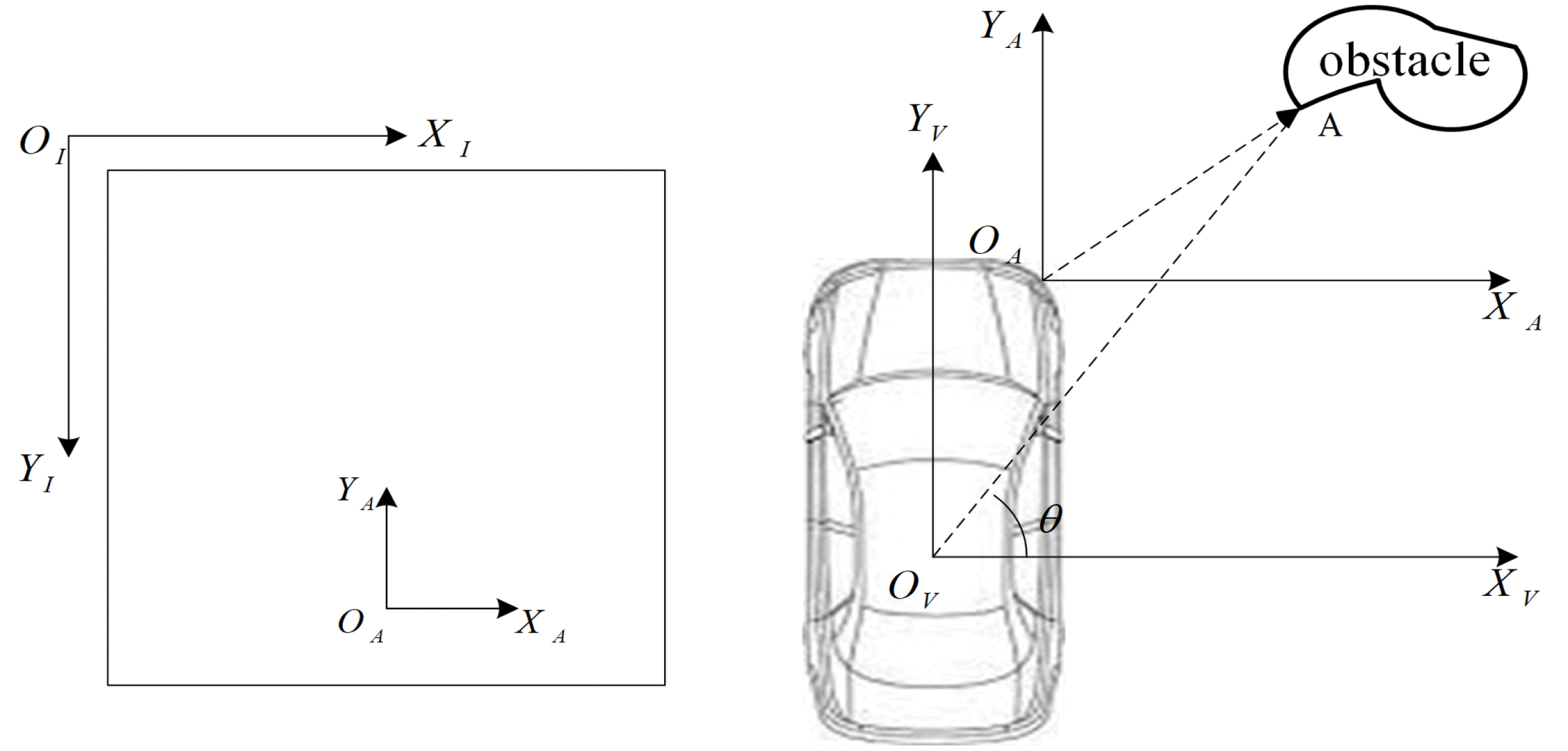
Coordinate system for obstacles state estimation via NOD-AVM: (A) ICS-ACS relationship; (B) ACS-VCS transformation.

In intelligent vehicle applications, the position of an obstacle relative to the ego-vehicle is critical for optimizing path planning, executing control strategies, and issuing warnings. Consequently, determining the precise location of detected obstacles is essential. In this study, the location of an obstacle is defined as the position of its closest point to the ego-vehicle. Thus, the objective of this section is to compute the coordinates of this closest point on the obstacle. To better illustrate obstacle localization estimation, **Fig 5** establishes three coordinate systems: the image coordinate system $X_{I}-O_{I}-Y_{I}$ (ICS), the NOD-AVM coordinate system $X_{A}-O_{A}-Y_{A}$ (ACS), and the vehicle coordinate system $X_{V}-O_{V}-Y_{V}$ (VCS).

As defined, the obstacle’s location corresponds to the coordinates of its closest point in the VCS, as shown in Fig 5B. Fig 5A depicts the relationship between ICS and ACS. Given that the driving ground is modeled as a plane, the ICS-to-ACS relationship can be represented by a two-dimensional transformation. Let $\left(x_{I},y_{I}\right)$ denote the closest obstacle point in the ICS, and $\left(x_{0},y_{0}\right)$ represent the ACS origin coordinate in ICS. The coordinates of any point $\left(x_{I},y_{I}\right)$ in ACS can be derived as follows:


$$\{\begin{array}{c} {}*20cx_{A}=(x_{I}-x_{0})s\\ y_{A}=(y_{0}-y_{I})s \end{array}$$ (11)

where $\left(x_{A},y_{A}\right)$ is the corresponding coordinate of $\left(x_{I},y_{I}\right)$ in ACS. s is the scale factor obtained by calibration.

Fig 5(b) illustrates the transformation between the ACS and VCS. Given that their coordinate axes are parallel, conversion between these systems requires only translational transformation, expressed as:


$$\{\begin{array}{c} {}*20cx_{V}=x_{A}+x_{1}\\ y_{V}=y_{A}+y_{1} \end{array}$$ (12)

where $\left(x_{A},y_{A}\right)$ denotes the obstacle’s coordinates in VCS, and $\left(x_{1},y_{1}\right)$ represents the ACS origin coordinates in the VCS, which can be obtained through calibration. For isolation fences or curbstones, these distances quantify the lateral offset between the ego-vehicle and the obstacles.

## 3. Experimental results and discussions

To validate the performance of the proposed method, real-world datasets were collected from two distinct scenarios in Wuhan, China. To ensure the reliability and accuracy of experimental outcomes, evaluation criteria are initially established, focusing on data accuracy, diversity, and distribution. Subsequently, the hardware and the sensor configurations are specified. Finally, comparative experiments with state-of-the-art methods are presented.

### 3.1 Evaluation criteria

Generally, an extracted bounding box is detected for obstacle detection, and IoU (Intersection of Union) is used for object classification. However, this scheme incurs high computation overhead. In contrast, this work focuses on detecting the presence of obstacles near the ego-vehicle without classification, significantly reducing computational demands. Our method is cost-efficient and covers all types of obstacles, not just cars or pedestrians. Detection results are sufficient to support high-level assisted-driving behaviors such as trajectory planning and collision avoidance. In extreme scenarios, the intelligent vehicle will remain stationary until the environmental safety is confirmed. Upon detection of an obstacle, our system utilizes the nearest point on the obstacle's surface to accurately represent its position. In cases where the obstacle is something like a fence or a curbstone, we describe its localization in terms of lateral distance.

### 3.2 Hardware setup and sensor calibration

**Fig 6:**
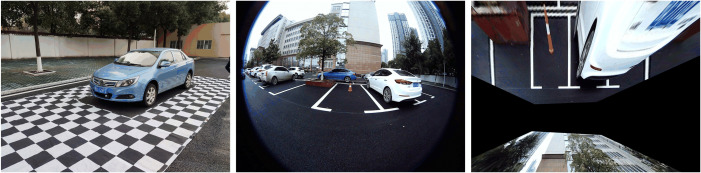
Calibration of the right wide-angle camera for IPM: (A) prototyped intelligent vehicle; (B) raw image; (C) IPM image.

To evaluate the performance of the proposed method, we implemented it on a PC equipped with an Intel(R) Core(TM)2 i7-7700HQ CPU 2.8GHz and 8GB RAM. The data acquisition platform is shown in Fig 6A. The prototyped intelligent vehicle is a BYD e5 pure-electric model fitted with a commercial AVM comprising four fisheye cameras mounted on the vehicle's each side. These wide-angle cameras include auxiliary lighting to enhance system robustness. Each camera captures raw images at a resolution of 1920 × 960 pixels, and all four cameras are synchronously triggered at 30 Hz to enhance temporal alignment. For clarity, detection results from the other three intersections are omitted. The cameras were pre-calibrated using Zhang's method beforehand [25]. Based on the calibration, we first undistort the raw images and then apply an IPM to project them onto the ground plane, as depicted in Fig 6C.

Our method's effectiveness was demonstrated through the analysis of datasets captured from real-world environments, specifically a campus parking area and an urban roadway scenario. On campus, we focused on detecting static obstacles commonly found in parking environments. In the urban scenario, we tested detection accuracy for pedestrians, vehicles, and safety infrastructure such as isolation fences. Correct detection of these obstacles is crucial for improving traffic safety. Our method is tailored for near-field obstacle detection and may be less effective at higher speeds due to motion blur and limited range. In line with related experiments, such as automatic parking systems, these functions are generally executed at low speeds. Accordingly, all experiments in our study were conducted below this threshold.

### 3.3 Experimental results on campus

**Fig 7:**
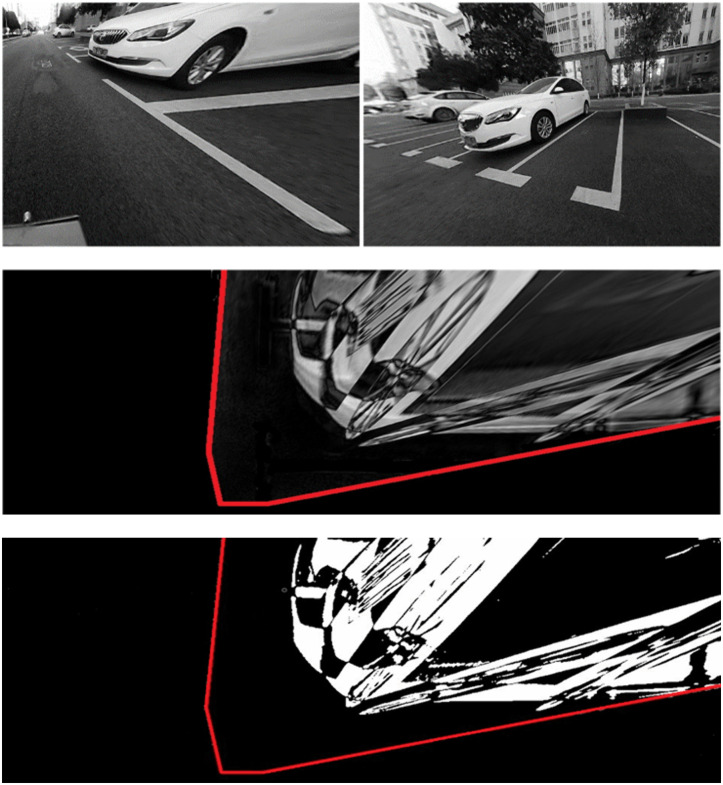
Results of obstacle detection on campus. (A) undistorted images; (B) IPM images; (C) detection results.

**Fig 8:**
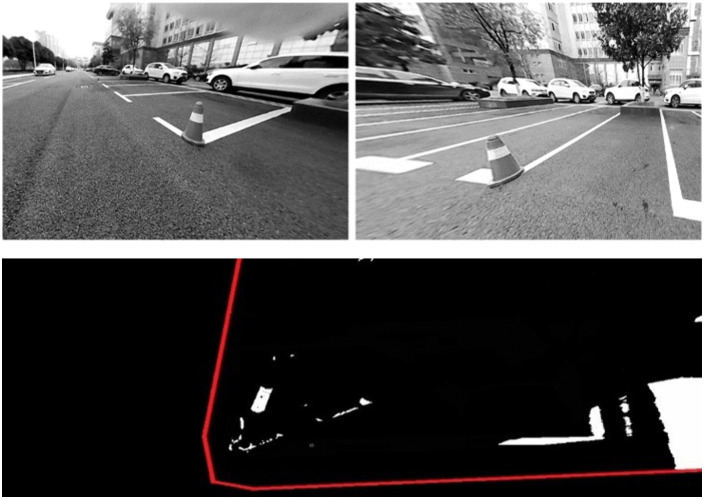
Detection result of a small obstacle (cone). (A) undistorted images; (B) detection results.

The first experiment was conducted on the Yujiatou campus of Wuhan University of Technology. The primary detection targets in this setting were parked vehicles and traffic cones, frequently encountered in daily driving scenarios. Using the homography matrix obtained in the previous section, we transformed the undistorted images into top-down views using IPM, projecting them onto the ground plane. As shown in Fig 7B, the raw IPM difference map—generated by subtracting sequential IPM images—contains significant noise. To remove this noise, thresholding and morphological operations were applied, resulting in a cleaner binary difference map as depicted in Fig 7C. The detection region corresponds to the overlapping areas among adjacent cameras. The experiments successfully detected both large obstacles (e.g. vehicles, see Fig 7) and small ones (e.g. cones, see Fig 8).

In the campus scenario, the prototype vehicle followed a predefined path and observed a total of 53 parked cars. The total length of the curbstone was approximately 200 meters, during which 61 images pairwise were collected. A total of 37 speed bumps and 33 traffic cones were also recorded. The proposed NOD-AVM was applied to detect the presence of obstacles following the previously described procedure. The detection results are presented in Table 1. Out of 184 detection instances, there were only 3 false detections, resulting in a detection accuracy of 98.36%. The false detections were primarily caused by shadows, which created visual similarities with the ground surface.

**Table 1:** Detection results of NOD-AVM on campus.

Types	Number	False Detection Number	Accuracy(%)
Vehicles	53	1	98.11
Curbstone	61	2	96.72
Speed bumps	37	0	100
Cones	33	0	100
**Total**	**184**	**3**	**98.36**

To evaluate the performance of the proposed method, it was compared with several state-of-the-art approaches, including 3D reconstruction based on stereo vision and the object detection using a monocular camera. The dataset for the method in [12] was collected using a ZED stereo camera concurrently with the dataset for NOD-AVM. The test dataset for the method in [24] was identical to that used for NOD-AVM. The method in [19] employed a monocular camera for depth estimation. Since the teacher model used in that method was trained on stereo image pairs collected using ZED, and these images were also used to retrain the model obtained from the author's GitHub. During testing, depth estimation was performed using real-time left images captured by ZED.

To evaluate the performance, three core performance metrics were quantified for systematic comparison with state-of-the-art approaches: detection accuracy, computational time, and localization accuracy. The quantitative comparison results are presented in Table 2. The proposed method achieved the best overall performance, with a detection accuracy of 98.36%, significantly higher than that of the others. The narrow 95% confidence interval further confirms the method's stability and reliability. In terms of computational efficiency, our approach required only 46ms per frame, demonstrating a substantial improvement over [12] (118ms), [19] (161ms), and [24] (238ms). This low latency enables real-time detection in practical applications. Whereas the other methods, especially [24], fall short of the temporal requirements for intelligent driving systems due to their high computational complexity. Regarding localization accuracy, our method exhibited an average error of only 1.20%, outperforming [12] (3.00%), [19] (10.60%), and [24] (10.20%). The superior precision mainly stems from the use of inverse perspective mapping and differential filtering, which effectively reduce geometric distortion and false detections near object boundaries. By contrast, the localization error in [19] and [24] is considerably larger, likely due to their dependence on coarse bounding-box or pixel-level disparity estimations that are less robust in near-field conditions. Method [12] showed the lowest accuracy among all due to its limited ability to reconstruct small obstacles and its relatively narrow stereo baseline, which affects depth precision. Overall, the proposed NOD-AVM achieves the optimal trade-off among accuracy, speed, and localization precision, demonstrating both high robustness and practical suitability for real-time near-obstacle detection in intelligent vehicle applications.

**Table 2:** Performance comparison with other state-of-the-arts detection methods.

Methods	Index	Mean Value	Standard Deviation	95%CI
Our method	DA (%)	98.36	4.92	(97.65,99.07)
	TC (ms)	46.00	2.30	(45.67,46.33)
	LA (%)	1.20	0.06	(1.19,1.21)
Method in [12]	DA (%)	87.62	4.38	(86.99,88.25)
	TC (ms)	118.00	5.90	(117.15,118.85)
	LA (%)	3.00	0.15	(2.98,3.02)
Method in [19]	DA (%)	93.41	4.67	(92.74,94.08)
	TC (ms)	161.00	8.05	(159.84,162.16)
	LA (%)	10.60	0.53	(10.52,10.68)
Method in [24]	DA (%)	92.33	4.62	(91.66,93.00)
	TC (ms)	238.00	11.90	(236.28,239.72)
	LA (%)	10.20	0.51	(10.13,10.27)

* DA = Detection Accuracy; TC = Time Consumption; LA = Localization Accuracy; CI = Confidence Interval.

### 3.4 Experimental results on the urban road

In contrast to the relatively controlled and predictable traffic within campus environments, which may feature elements like students, bicycles, and designated parking area, urban traffic conditions are significantly more complex. Urban roads are characterized by a diverse array of dynamic such as pedestrians, moving vehicles, and various static elements including isolation fences and traffic signals. In addition, driver blind spots further increase the risk to pedestrian safety, making reliable pedestrian detection a critical requirement for intelligent vehicle systems.

**Fig 9:**
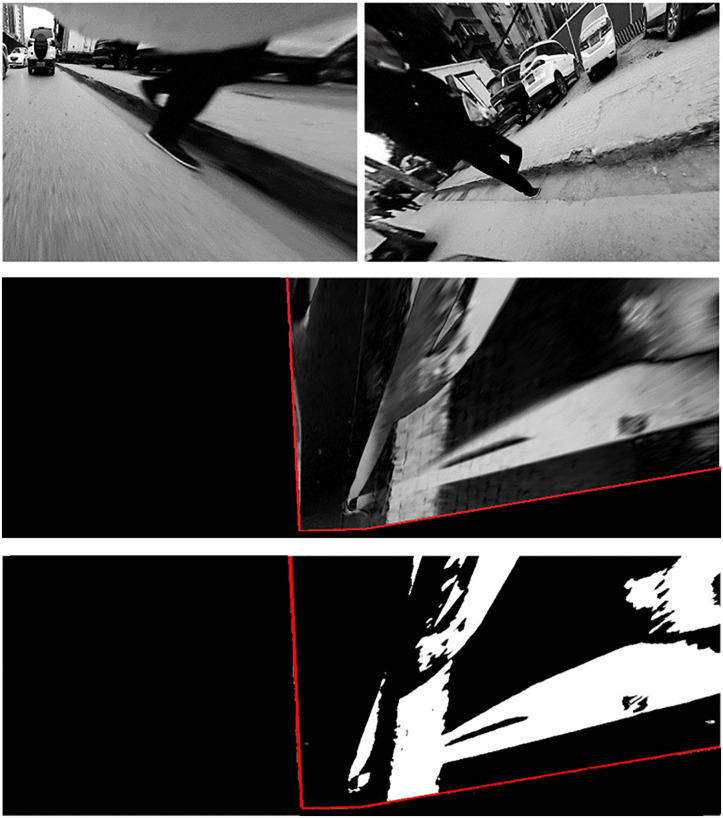
Detection results using NOD-AVM on urban road. (A) undistorted images; (B) IPM difference map; (C) detection results.

The pedestrian detection results are shown in Fig 9. As illustrated, the proposed method successfully detects a pedestrian within the intersection area. What's more, Fig 9C shows that the method is capable of identifying a sunken area in the road surface. Given that pothole detection is not the primary focus of this study, no further experiments were carried out in this area. Nevertheless, the results indicate significant potential for broader applications.

**Fig 10:**
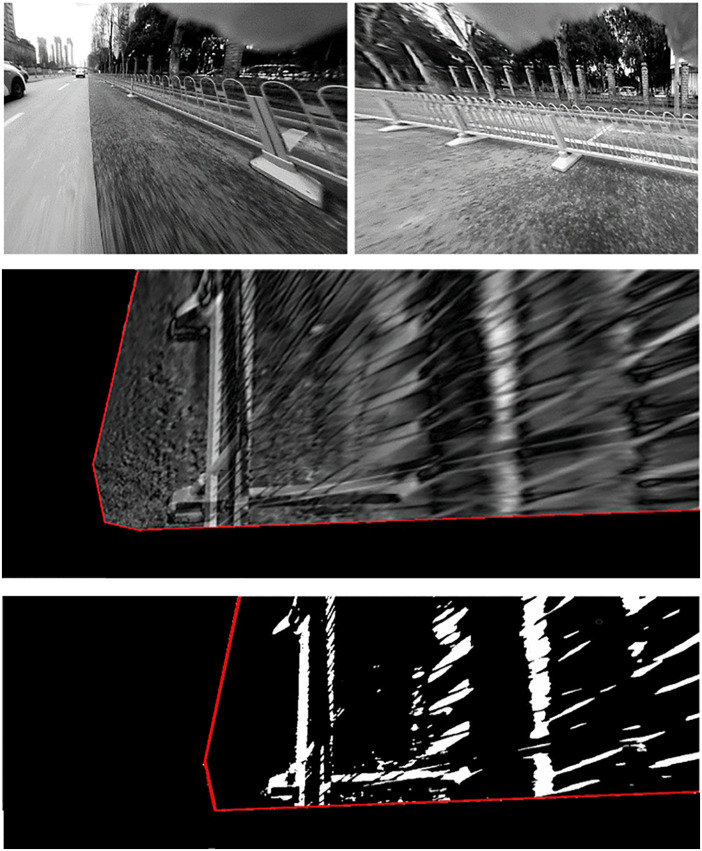
Detection result of isolation fence: (A) undistorted images, (B) IPM difference map, (C) detection results.

Isolation fences, commonly installed along the median of urban roads, are essential traffic control structures but can also result in traffic accidents if not properly detected. Therefore, the detection of isolation fences was also implemented, and the corresponding results are presented in **Fig 10**. The observed differences in the detection regions between the two experiments are attributed to variations in the camera installation positions. Therefore, detection of both moving pedestrians and isolation fences was carried out, as accurate identification of these obstacles is essential for ensuring driving safety. The data collection and implementation procedures were consistent with those in the campus experiments. The results of near obstacle detection in urban road scenarios are presented, demonstrating improved accuracy and robustness in challenging traffic conditions. As shown in Figs 7, 10, the proposed NOD-AVM method can be used to calculate the distance between the ego-vehicle and the detected obstacles. This distance can subsequently be applied to optimize driving paths or provide early warnings of potential hazards.

In urban scenarios, a total of 137 images pairwise were collected for obstacle detection. Among these, 28 were used for detecting moving vehicles, 24 for pedestrians, 64 for isolation fences, and 21 for coaches. The detection results for different obstacle types are summarized in Table 3, which provides a detailed inventory along with detection accuracy listed in the final column. The overall performance of the proposed near obstacle detection method in urban environment is summarized in the bottom row of Table 3.

**Table 3:** Detection results on urban roads.

Types	Number	False Detection Number	Accuracy(%)
Vehicles	28	0	100
Pedestrian	24	1	95.83
Isolation fence	64	0	100
Coaches	21	0	100
Total	**137**	**1**	**99.27**

The results demonstrate that the proposed NOD-AVM remains effective and practical in urban environments, exhibiting similarly excellent performance to that observed in campus experiments. The NOD-AVM method proved to be both robust and efficient in detecting various types of obstacles. In urban traffic scenarios, the rapid movement of obstacles poses a significant challenge for accurate detection and measurement of distances to ego-vehicles. As a result, precise localization data could not be reliably collected, and localization accuracy is therefore not reported.

## 4. Conclusions and future work

This paper proposes a near obstacle detection method based on an off-the-shelf AVM, designed to compensate for the blind sensing areas of conventional vehicle sensors. In our approach, raw images are undistorted using intrinsic matrix and then projected onto the ground plane through IPM with extrinsic calibration. Obstacle detection and localization are subsequently achieved via segmentation and filtering of the IPM difference map under the ground-plane assumption. The proposed NOD-AVM relies on fundamental image processing techniques, such as normalization and image difference, enabling accurate and reliable detection without requiring time consuming 3D reconstruction or computationally intensive training procedures. Experiments on real-world campus and urban road datasets demonstrate that NOD-AVM can effectively detect a variety of static and dynamic obstacles within a range of 5.2 meters longitudinally and 6 meters laterally, with a field of view of approximately 75 degrees. These results indicate that the method is promising for scenarios where computational resources are constrained and lightweight solutions are preferred.

Despite its effectiveness, the method also faces certain limitations. First, the assumption of a flat road surface may not always hold: vehicle roll and pitch changes during acceleration or braking, or driving on slopes and uneven roads, can degrade calibration accuracy. To address this, future work will explore a joint optimization framework for vehicle pose and camera extrinsic parameters, supported by high-definition maps and graph-based refinement. Second, detection performance may decline when obstacles have low contrast with the background or are transparent, which are inherently difficult to distinguish. Integrating multi-source heterogeneous sensors (e.g., LiDAR, radar) could help overcome these challenges by leveraging complementary sensing modalities.

In terms of generalization, if a vehicle platform is equipped with an AVM system, extrinsic calibration can be performed using a checkerboard pattern. For platforms without an AVM system, the same functionality can be achieved with independently installed wide-angle cameras. Moreover, performance under adverse environmental conditions such as rain, low light, or shadows could be improved by integrating robust preprocessing techniques, including de-noising filters, contrast enhancement, shadow removal, and weather-invariant image features. Overall, while comparative evaluation against state-of-the-art methods was limited in scope, this study demonstrates that NOD-AVM provides a practical and efficient solution for near-obstacle detection. Future research will focus on broader benchmarking, diverse traffic scenarios, and sensor fusion to further enhance its robustness and generalization.
